# Comparison of Rates of Overdose and Hospitalization After Initiation of Medication for Opioid Use Disorder in the Inpatient vs Outpatient Setting

**DOI:** 10.1001/jamanetworkopen.2020.29676

**Published:** 2020-12-15

**Authors:** Jake R. Morgan, Joshua A. Barocas, Sean M. Murphy, Rachel L. Epstein, Michael D. Stein, Bruce R. Schackman, Alexander Y. Walley, Benjamin P. Linas

**Affiliations:** 1 Department of Health Law, Policy, and Management, Boston University School of Public Health, Boston, Massachusetts; 2 Section of Infectious Diseases, Department of Medicine, Boston Medical Center, Boston, Massachusetts; 3 Department of Population Health Sciences, Weill Cornell Medical College, New York, New York; 4 Section of General Internal Medicine, Department of Medicine, Boston Medical Center and Boston University School of Medicine, Boston, Massachusetts; 5 Department of Epidemiology, Boston University School of Public Health, Boston, Massachusetts

## Abstract

**Importance:**

Whereas outpatient treatment with medication for opioid use disorder (MOUD) is evidence based, there is a large network of inpatient facilities in the US that are reimbursed by commercial insurers and do not typically offer MOUD.

**Objective:**

To compare the rates of opioid-related overdose and all-cause hospitalization after outpatient MOUD treatment vs inpatient care.

**Design, Setting, and Participants:**

This comparative effectiveness research study used deidentified claims of commercially insured individuals in the US from the MarketScan Commercial Claims and Encounters Database from January 1, 2010, to December 31, 2017, to obtain a sample of 37 090 individuals with opioid use disorder who initiated treatment with inpatient care and/or MOUD. Data were analyzed from October 1, 2019, to May 1, 2020. To address nonrandom treatment assignment, individuals with opioid use disorder who initiated MOUD or who entered inpatient care were matched 1:1 based on propensity scores.

**Exposures:**

The independent variable of interest was the type of treatment initiated. Individuals could initiate 1 of 5 potential treatments: (1) outpatient MOUD, (2) short-term inpatient care, (3) short-term inpatient care followed by outpatient MOUD within 30 days, (4) long-term inpatient care, or (5) long-term inpatient care followed by outpatient MOUD within 30 days.

**Main Outcomes and Measures:**

Opioid-related overdose and all-cause hospitalization at any point within the 12 months after treatment of opioid use disorder. The hazard for each outcome was estimated using a time-to-event Cox proportional hazards regression model.

**Results:**

The cohort included 37 090 individuals matched 1:1 between inpatient and outpatient treatment (20 723 [56%] were younger than 30 years; 23 250 [63%] were male). After propensity score matching, compared with the inpatient treatments, initiation of outpatient MOUD alone was followed by the lowest 1-year overdose rate (2.2 [95% CI, 2.0-2.5] per 100 person-years vs 3.5 [95% CI, 2.7-4.4] to 7.0 [95% CI, 4.6-10.7] per 100 person-years) and hospitalization rate (39 [95% CI, 38-40] per 100 person-years vs 57 [95% CI, 54-61] to 74 [95% CI, 73-76] per 100 person-years). Outpatient MOUD was also associated with the lowest hazard of these events compared with inpatient care, which had hazard ratios ranging from 1.71 (95% CI, 1.35-2.17) to 2.67 (95% CI, 1.68-4.23) for overdose and 1.33 (95% CI, 1.23-1.44) to 1.90 (95% CI, 1.83-1.97) for hospitalizations.

**Conclusions and Relevance:**

The results of this comparative effectiveness research study suggest that lower rates of subsequent overdose and hospitalization are associated with outpatient MOUD compared with short- or long-term inpatient care. When patients and clinicians have a choice of treatment, outpatient MOUD treatment may be associated with lower overdose and hospitalization on balance. Future research should assess which patients benefit most from inpatient care and how best to leverage existing inpatient treatment infrastructure.

## Key Points

**Question:**

Do the rates of overdose and hospitalization differ after outpatient medication treatment or inpatient care for opioid use disorder?

**Findings:**

In this comparative effectiveness research study of 37 090 propensity score–matched individuals with opioid use disorder receiving outpatient medication treatment or inpatient care, all forms of inpatient care (short or long term) were associated with higher risk of subsequent overdose and hospitalization.

**Meaning:**

The findings suggest that outpatient medication may be less likely than inpatient care to be associated with subsequent overdose or hospitalization.

## Teaser

This comparative effectiveness research study uses 2010-2017 data from the MarketScan Commercial Claims and Encounters Database to compare the rates of opioid-related overdose and all-cause hospitalization after treatment of opioid use disorder in outpatient vs inpatient care settings.

## Introduction

The opioid overdose crisis continues to be a major public health challenge, and effective treatment remains underused.^1^ Medication for opioid use disorder (MOUD) delivered in an outpatient setting has been shown to be associated with improved health outcomes, but its use is limited by access and retention challenges.^1,2,3^ Inpatient addiction care approaches primarily include managed withdrawal and supportive care but can and should include transition to outpatient MOUD^4^; however, fewer than half (approximately 38%) offer MOUD beyond withdrawal management,^5,6^ and relapse among those who are not inducted onto MOUD is common.

Few studies have directly compared the outcomes after initiation of MOUD in an outpatient setting vs initiating inpatient care,^7^ and none, to our knowledge, have directly compared the association between the timing of outpatient MOUD (after inpatient care or as a stand-alone outpatient service) and our outcomes of overdose and hospitalization. When individuals receiving outpatient MOUD and inpatient care have been compared, those receiving outpatient MOUD have had fewer overdose and opioid-related acute care events in the 3 or 12 months after initiation,^7^ a lower risk of mortality during a 12-month period,^8^ and lower rates of relapse to opioid use.^9^ An important challenge in conducting this research is to consider who receives each type of care because patient and clinician preferences and care access vary, and treatment assignment is not random.^10,11^ This introduces selection bias and confounding by indication, which makes conducting comparative studies challenging.

We addressed this evidence gap by comparing the incidence of opioid-related overdose (directly associated with opioid use disorder [OUD]) and all-cause hospitalization (a broader health outcome) after treatment with either outpatient MOUD or inpatient care strategies using propensity score methods to address nonrandom assignment. We believe that these findings will be relevant to clinicians, patients, and insurers evaluating treatment options for OUD.

## Methods

### Data

For this comparative effectiveness research study, we identified a cohort of individuals aged 13 years or older with OUD who initiated inpatient care and/or MOUD and were included in the IBM-Watson MarketScan Commercial Claims and Encounters Database (MarketScan) from January 1, 2010, to December 31, 2017. MarketScan is an insurance claims–based data set that includes ambulatory and inpatient visits, laboratory and diagnostic testing, and outpatient pharmacy claims from a nationally representative sample of the commercially insured population in the US.^12^ The Boston University Institutional Review Board deemed this study as not human studies research and thus exempt from review. The study followed the International Society for Pharmacoeconomics and Outcomes Research (ISPOR) reporting guidelines.

### Cohort Definition

Inclusion criteria included (1) a diagnosis of OUD in medical claims, (2) claims for either an inpatient OUD admission or outpatient MOUD, and (3) outpatient pharmacy insurance coverage. Inpatient care included both short-term admissions (typically 3 to 7 days) to intensive treatment facilities such as detoxification units and long-term admissions (ranging from 10 days to several months) to stabilization units and supportive living environments in which OUD was listed as the reason for admission. We identified inpatient care through revenue codes and the Healthcare Common Procedure Coding System (eAppendix in the Supplement). We reviewed these codes with a clinical addiction medicine expert (A.Y.W.) and verified them by comparing the median length of stay for short-term care (5 days) and long-term care (14 days). To identify inpatient care or MOUD initiation, we identified the first episode that was preceded by at least 12 months during which neither inpatient care nor MOUD treatment was observed. We further identified whether inpatient episodes were followed by medication treatment within 30 days. MOUD included prescriptions for naltrexone (oral or injectable) and buprenorphine (monoformulated or coformulated with naloxone) and were identified from a filled prescription. For MOUD treatment, we required an OUD diagnosis within 3 months before the first medication prescription fill to exclude individuals prescribed medications for other conditions (eg, oral naltrexone is also prescribed for alcohol use disorder). Methadone maintenance treatment is not routinely captured in commercial claims data, so we were unable to identify individuals receiving methadone for OUD. We were not able to identify events prompting treatment but assessed recent overdose before treatment as a measure of OUD severity and a possible treatment pathway.

### Outcome Measures

We considered 2 primary outcomes: opioid-related overdose and all-cause hospitalization. Overdose events were identified based on *International Classification of Diseases, Ninth Revision* and *International Statistical Classification of Diseases and Related Health Problems, Tenth Revision* codes in inpatient or outpatient medical claims,^13^ and hospitalizations were identified by recorded inpatient admissions after initiation of inpatient care or MOUDs.

### Addressing Selection Bias

We used a propensity score matching technique to address selection bias. The propensity score represents the likelihood of being assigned to a particular treatment group and is a function of observed characteristics that are believed to affect treatment assignment. On the basis of existing literature, we identified demographic and clinical characteristics associated with treatment assignment, even though these treatment assignments were not best clinical practice. For example, younger (compared with older) adults and those covered by a parent’s insurance plan may be more likely to receive inpatient care based on the perception that MOUD is particularly undesirable for youths.^14^ A similar preference for inpatient care may also exist among those with comorbid mental health conditions.^15^ These factors have also been shown to be independently associated with negative sequelae of OUD, such as overdose.^13^

We matched individuals who initiated outpatient MOUD treatment and inpatient care 1:1 based on propensity scores. We included the following demographic and clinical factors in the propensity score estimates: age, evidence of a naloxone prescription in the 90 days before treatment, type of commercial insurance, whether the individual was the insurance holder or a covered dependent, year of treatment, region of residence (defined by whether an individual lives in the Northeast, Midwest, South, or West of the US), modified Elixhauser comorbidity score (excluding drug and alcohol use, which are captured separately), evidence of an overdose in the 90 days before treatment, and evidence of concurrent nonopioid substance use disorder at the time of treatment initiation, including alcohol, amphetamine, cannabis, cocaine, hallucinogen, and sedative use disorders. We dichotomized age as 30 years or older vs younger than 30 years based on literature suggesting an association of the transition from emerging and young adulthood with OUD outcomes.^16,17^ We further required a match on sex. We used a random order, greedy matching algorithm with a caliper of 0.20.^18^ Details of the matching algorithm can be found in the eAppendix in the Supplement. To assess the robustness of the propensity score matching procedure, we compared the prematching and postmatching standardized mean differences between the outpatient MOUD treatment and inpatient care cohorts. Whereas we analyzed inpatient care groups separately (short term vs long term and with or without medication treatment within 30 days of discharge), we matched on the decision to initiate medication treatment or inpatient care based on clinical intuition and the clustering of prematch characteristics (eTable 2 in the Supplement). We conducted a sensitivity analysis to evaluate alternative matching strategies, including matching on individual inpatient categories, and found that the resulting matches were worse and did not change the results (eTable 1 in the Supplement).

### Statistical Analysis

We conducted a 2-step analysis of the propensity score–matched cohort. First, we compared the incidence rates for each outcome during a 1-year period after initiation of the given care category (MOUD vs inpatient care). Second, we assessed how the care initiation category was associated with time to first overdose or hospitalization and used a multivariable Cox proportional hazards regression model to incorporate differential timing of outcome and follow-up length (the median time to each outcome after treatment was 220 days [interquartile range, 143 days] for opioid-related overdose and 129 days [interquartile range, 295 days] for all-cause hospitalization). From this model, we estimated a hazard ratio (HR) comparing the rate of overdose or hospitalization between outpatient MOUD treatment and the categories of inpatient care (ie, short-term vs long-term). Individuals were censored when they experienced the outcome, switched treatment modalities, were no longer enrolled in a participating plan, or at study end (December 31, 2017). We included a set of demographic and clinical variables similar to those used for matching in the multivariable model to test their association with the outcome independent from their association with treatment selection. Including these variables in the matched analysis allowed us to identify their independent association with overdose and hospitalization after nonrandom treatment selection bias had been addressed by the propensity score matching.^19,20^ Analyses were conducted from October 1, 2019, to May 1, 2020, using SAS, version 9.4 (SAS Institute Inc). Statistical significance was set at *P* < .05 using a 2-tailed test.

We calculated E-values to assess the potential extent of residual confounding remaining after matching. E-values estimate how large an unmeasured confounder between the treatment exposure and outcomes would need to be for such a confounder to explain away any association found between exposure and outcome.^21^

We conducted a sensitivity analysis disaggregating the combined MOUD exposure from its component medications (buprenorphine, oral naltrexone, and injectable naltrexone) in the Cox proportional hazards regression model. The results allowed us to assess whether the difference between MOUD and inpatient care was associated with the type of MOUD.

## Results

We identified a total of 46 524 individuals who initiated MOUDs and 24 212 individuals who initiated inpatient care; the propensity score–matched sample included 37 090 total individuals (20 723 [56%] were younger than 30 years, and 23 250 [63%] were male) balanced equally between outpatient MOUD initiations and inpatient admissions. Among inpatient admissions, 12 628 were short-term stays alone; 3683 were short-term admissions followed by outpatient MOUD within 30 days; 1921 were long-term admissions alone; and 313 were long-term admissions followed by outpatient MOUD within 30 days. Table 1 shows the postmatching characteristics of individuals initiating outpatient MOUD treatment and those initiating inpatient care. The standardized mean differences comparing the prematch and postmatch samples revealed successful balancing on the matched variables (eAppendix in the Supplement); eTable 3 in the Supplement details the characteristics of the unmatched individuals.

**Table 1.** Characteristics of Individuals Initiating Outpatient Medication for Opioid Use Disorder or Inpatient Care After Propensity Score Matching

Characteristic	Patients, No. (%)	
	Initiated outpatient medication treatment (n = 18 545)	Initiated inpatient care (n = 18 545)
Sex		
Male	11 625 (62.7)	11 625 (62.7)
Female	6920 (37.3)	6920 (37.3)
Age, y		
<30	10 591 (57.1)	10 132 (54.6)
≥30	7954 (42.9)	8413 (45.4)
Insurance		
PPO	11 069 (59.7)	11 281 (60.8)
HMO	2162 (11.7)	2024 (10.9)
POS	1471 (7.9)	1468 (7.9)
Other	3843 (20.7)	3772 (20.3)
US region		
Northeast	4572 (24.7)	4234 (22.8)
Midwest	3823 (20.6)	3810 (20.5)
South	6307 (34.0)	6874 (37.1)
West	3739 (20.2)	3511 (18.9)
Unknown	104 (0.6)	116 (0.6)
Insurance coverage		
Primary holder	5705 (30.8)	6069 (32.7)
Spouse	3659 (19.7)	3792 (20.4)
Dependent	9181 (49.5)	8684 (46.8)
Modified Elixhauser score^a^		
0	7507 (40.5)	8042 (43.4)
1	5856 (31.6)	5723 (30.9)
2	2843 (15.3)	2681 (14.5)
≥3	2339 (12.6)	2117 (11.4)
Retail pharmacy naloxone prescription^b^		
Yes	331 (1.8)	344 (1.9)
No	18 214 (98.2)	18 201 (98.1)
Concurrent substance use at initiation^c^		
Alcohol^d^	3190 (17.2)	3143 (16.9)
Amphetamines	638 (3.4)	737 (4.0)
Marijuana	1666 (9.0)	1679 (9.1)
Cocaine	871 (4.7)	945 (5.1)
Hallucinogens	59 (0.3)	72 (0.4)
Sedatives	1669 (9.0)	2069 (11.2)
≥1 Overdose before initiation^c^		
Yes	355 (1.9)	338 (1.8)
No	18 190 (98.1)	18 207 (98.2)

Abbreviations: HMO, health maintenance organization; POS, point of service; PPO, preferred provider organization.

^a^ The Elixhauser score was modified to exclude drug and alcohol use because those are included separately.

^b^ Any time in the 90 days before treatment initiation.

^c^ Diagnosis code in the 90 days before first initiation.

^d^ Oral naltrexone initiations were excluded when alcohol use disorder was present within 90 days prior.

The Figure shows the opioid-related overdose and all-cause hospitalization rates among individuals receiving MOUD and inpatient care strategies. Individuals who initiated outpatient MOUD treatment alone had the lowest rate of opioid-related overdose in the following year of all treatments (2.2; 95% CI, 2.0-2.5 per 100 person-years). Those entering care in an inpatient setting had a higher rate of opioid-related overdoses in every case, from 3.5 per 100 person-years (95% CI, 2.7-4.4 per 100 person-years) to 7.0 per 100 person-years (95% CI, 4.6-10.7 per 100 person-years).

**Figure.**
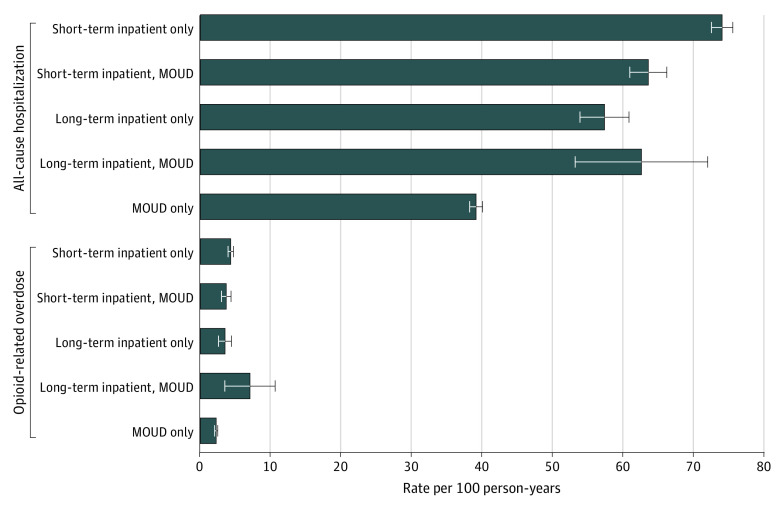
One-Year Rates of Overdose and Hospitalization Among Individuals Initiating Medication for Opioid Use Disorder (MOUD) or Inpatient Care Error bars indicate 95% CIs. eTable 6 in the Supplement gives detailed results.

Individuals who initiated outpatient MOUD alone also had a lower rate of all-cause hospitalization in the year following treatment compared with all other treatments (39 per 100 person-years; 95% CI, 38-40 per 100 person-years). Those initiating inpatient care had up to nearly double the hospitalization rate, ranging from 57 per 100 person-years (95% CI, 54-61 per 100 person-years) to 74 per 100 person-years (95% CI, 73-76 per 100 person-years).

We estimated the HR describing the risk of an individual experiencing the outcome at any given time for each variable compared with those initiating outpatient MOUD treatment alone (reference group) using a Cox proportional hazards regression model. The findings for time to first opioid-related overdose and to first hospitalization were similar (Table 2). All inpatient care strategies were associated with an increased risk of opioid-related overdose, often twice the risk of the MOUD reference groups, from 1.71 (95% CI, 1.35-2.17) to 2.67 (95% CI, 1.68-4.23). A post hoc Wald test found no difference in the association with overdose among the inpatient care groups. All-cause hospitalization HRs were also increased for inpatient categories compared with outpatient MOUD, ranging from 1.33 (95% CI, 1.23-1.44) to 1.90 (95% CI, 1.83-1.97); short-term inpatient care alone was associated with the highest hazard of the group.

**Table 2.** Time to Overdose and Hospitalizations Among Individuals Initiating Treatment of Opioid Use Disorder

Variable^a^	Hazard ratio (95% CI)	
	Opioid-related overdose	All-cause hospitalization
Treatment initiation		
MOUD	1 [Reference]	1 [Reference]
Short-term inpatient	2.23 (1.97-2.52)	1.90 (1.83-1.97)
Short-term inpatient followed by MOUD	2.08 (1.75-2.47)	1.74 (1.64-1.84)
Long-term inpatient	1.71 (1.35-2.17)	1.33 (1.23-1.44)
Long-term inpatient followed by MOUD	2.67 (1.68-4.23)	1.16 (0.96-1.42)
Sex		
Male	1 [Reference]	1 [Reference]
Female	0.95 (0.85-1.07)	1.18 (1.14-1.22)
Age, y		
<30	[Reference]	[Reference]
≥30	0.49 (0.38-0.63)	0.87 (0.80-0.93)
Insurance coverage		
Primary holder	1 [Reference]	1 [Reference]
Spouse	0.84 (0.67-1.05)	1.12 (1.06-1.18)
Dependent	1.65 (1.30-2.08)	1.51 (1.41-1.63)
Modified Elixhauser score^b^		
0	1 [Reference]	1 [Reference]
1	1.06 (0.93-1.20)	1.13 (1.09-1.18)
2	1.32 (1.11-1.57)	1.36 (1.29-1.44)
≥3	1.45 (1.18-1.79)	1.94 (1.83-2.06)
Retail pharmacy naloxone prescription		
Yes	1.95 (1.51-2.52)	1.74 (1.57-1.92)
No	1 [Reference]	1 [Reference]
Concurrent substance use at initiation^c^		
Alcohol^d^	0.91 (0.77-1.08)	1.11 (1.06-1.17)
Amphetamines	0.99 (0.73-1.35)	1.14 (1.04-1.25)
Marijuana	0.93 (0.79-1.10)	1.01 (0.95-1.07)
Cocaine	1.28 (1.02-1.61)	1.19 (1.11-1.29)
Hallucinogens	0.90 (0.40-2.01)	1.12 (0.87-1.44)
Sedatives	1.30 (1.09-1.54)	1.11 (1.05-1.17)
≥1 Overdose before initiation		
Yes	3.29 (2.64-4.09)	1.21 (1.08-1.35)
No	1 [Reference]	1 [Reference]

Abbreviation: MOUD, medication for opioid use disorder.

^a^ Each model simultaneously adjusted for all included variables. We also controlled for region of residence, type of commercial insurance, and year of treatment initiation. Full results are available in eTable 5 in the Supplement. Sex, age, and insurance coverage were measured at the time of initial treatment. Elixhauser score, pharmacy naloxone, concurrent substance use, and overdose before initiation of treatment were measured using data from 90 days before initiation.

^b^ The Elixhauser score was modified to exclude drug and alcohol use because those are included separately.

^c^ Substance use variables are dichotomous (reference is no evidence of use) and overlapping.

^d^ Those with evidence of alcohol use disorder were not eligible for inclusion in the oral naltrexone cohort.

Individuals aged 30 years or older were almost half as likely to experience an opioid-related overdose at any time (HR, 0.49; 95% CI, 0.38-0.63) and 13% less likely to experience hospitalization (HR, 0.87; 95% CI, 0.80-0.93). Those who were covered as dependents rather than primary insurance plan holders were at greater risk for both outcomes (HR, 1.65 [95% CI, 1.30-2.08] for opioid-related overdose; HR, 1.51 [95% CI, 1.41-1.63] for hospitalization). That is, young adults were overall less likely to experience a negative outcome compared with older adults, but young adults who were a dependent rather than a primary plan beneficiary had increased risk of a negative outcome.

E-values were large compared with the included model confounders for both outcomes (eAppendix in the Supplement). On this basis of the findings, the literature, and our clinical expertise, we believe it is unlikely that an unmeasured or unknown confounder would have a stronger association with the outcomes than the known risk factors that we included.^22^

In the sensitivity analysis, of the 18 545 individuals initiating MOUD, 16 043 (87%) initiated buprenorphine, 1300 (7%) initiated oral naltrexone, and 1202 (6%) initiated injectable naltrexone. In the Cox proportional hazards regression model disaggregating MOUD by type of medication, we did not detect a statistically significant difference among the medication types, and results still indicated that inpatient treatments were associated with an increased risk of each outcome (eTable 4 in the Supplement).

## Discussion

This comparative effectiveness research study compared the incidence of opioid-related overdose and all-cause hospitalization after outpatient treatment with MOUD alone with initiation of care for opioid dependence in an inpatient setting. In the year after initiation, those treated with outpatient MOUD alone had fewer hospitalizations and overdoses than persons seeking inpatient care initially, with or without aftercare MOUD. When examining hospitalization after inpatient care, we found a dose effect for inpatient treatment. Hospitalization was less common after a long-term stay than a short-term admission and was less common when a short-term stay was followed by MOUD than when it was not. These findings suggest that among inpatient care strategies, longer stays may be more beneficial than short stays but that the effect of MOUD is limited to short-term stays and may not be as beneficial after extended interaction with inpatient care. We did not observe this association in our model of overdose. The conclusions are limited to the population represented in the matched sample. The unmatched sample of individuals who initiated MOUD had a low prevalence of concurrent nonopioid substance use (eTable 3 in the Supplement). More research is needed to fully explore treatment outcomes in this group.

The finding that initiating MOUD alone was associated with better outcomes compared with inpatient treatment, even when inpatient treatment was followed by MOUD, suggests that a multistep approach to initiating MOUD may present barriers that are associated with decreased efficacy of MOUD. In a previous study,^23^ transition periods following inpatient care were associated with elevated risk for overdose; this association may be present in any transition from inpatient care, even to MOUDs, and warrants further study. Although we did not examine nonmedication outpatient treatment in this study, other work has found that medication alone is preferred to psychosocial treatment alone,^7,9^ and the current study adds to a broad evidence base supporting outpatient MOUD as the first-line treatment.

We assessed the association of treatment with other demographic and clinical variables to provide useful information for clinicians initiating treatment in either setting. More research is needed to explain the finding that dependents were at greater risk of overdose or hospitalization. This may partly be explained by more severe cases among those with dependent coverage given that these individuals may not be employed and maintaining their own employee-based health coverage and may also indicate parental involvement in health care decision-making.

### Strengths and Limitations

This study has strengths. The inclusion of a large data set allowed us to examine different combinations of MOUD and inpatient care, and we used a method to control for nonrandom assignment to outpatient vs inpatient treatment. The propensity score algorithm was designed a priori according to findings from the relevant addiction literature and included potential factors associated with treatment regardless of effect size because previous research has shown that including any variables associated with treatment will reduce selection bias more than it increases variance.^24^

This study has limitations. As with all retrospective analyses, it is possible that some unmeasured confounding persists. Although we attempted to address this confounding with a matching approach, we were only able to match on observable characteristics, and persistent, unobserved differences in treatment selection might bias the results. However, the calculation of E-values showed that an unmeasured confounder would need to have an association with outcomes that was substantially larger than the measured association of any factor included in the model to completely mitigate the findings. Moreover, although we did not know why individuals sought treatment or the severity of their disease, we included recent opioid-related overdose before treatment to capture recent health care use owing to OUD. Although unmeasured confounding is an important limitation to any observational study comparing approaches to OUD care, we believe this and other studies are critical to expand methodologic approaches for observational data given that a randomized clinical trial seeking to answer this same question would be unethical and infeasible. Because we used an administrative claims data set, we were not able to assess relapse to drug use or reliably identify deaths, and individuals were censored when they switched insurers. Thus, we could not measure whether an overdose or hospitalization was fatal. In addition, because commercial insurance during the study period did not cover methadone, a common MOUD, we were unable to capture either the initiation of methadone alone or the initiation of methadone shortly after inpatient discharge. Furthermore, because the data only included outpatient medication fills, we were not able to observe MOUD use in inpatient settings, such as when buprenorphine is used to taper an individual off opioids or MOUD is used in conjunction with inpatient care. However, the literature indicates that MOUD in these settings is typically used to manage withdrawal rather than serve as initiation for long-term treatment.^5,6^ We did observe inpatient programs that successfully linked individuals to outpatient treatment in the category of inpatient care plus MOUD initiation within 30 days.

## Conclusions

The findings of this study suggest that when patients and clinicians have a choice between outpatient MOUD initiation and a period of inpatient care that might or might not lead to outpatient MOUD use, choosing outpatient MOUD is less likely to be associated with subsequent overdose or hospitalization than is initiating inpatient care. Even when treatment options are limited (eg, in responding to emergency overdose situations), these results suggest the benefit of a treatment infrastructure focused on MOUD. This research adds to the increasing evidence that inpatient care may be associated with more overdoses and hospitalizations than outpatient MOUD treatment of individuals with OUD and suggests that extended inpatient stays and initiation of MOUD after inpatient discharge may be associated with reduced hospitalizations compared with shorter inpatient stays without subsequent MOUD uptake. Whereas more research is needed to assess whether certain subgroups benefit more from inpatient care and how to best use the existing inpatient care infrastructure, increasing capacity for outpatient MOUD treatment appears to be an evidence-based option for a national response to the opioid overdose epidemic.

## Notes

Supplement. **eAppendix.** Defining inpatient treatment and propensity score matching approach **eFigure.** Comparing standardized mean difference before and after matching **eTable 1.** Summary of baseline and alternative propensity score matching strategies **eTable 2.** Cohort characteristics prior to propensity score matching **eTable 3.** Characteristics of unmatched individuals **eTable 4.** Expanded Cox proportional hazards regression model output **eTable 5.** Cox proportional hazards model with disaggregated medication categories **eTable 6.** Detailed results of 1-year outcome incidence rates **eReferences**
