# Supplementary material for: Comparison of Rates of Overdose and Hospitalization After Initiation of Medication for Opioid Use Disorder in the Inpatient vs Outpatient Setting
Source: JAMA Netw Open. 2020 Dec 15;3(12):e2029676. doi: 10.1001/jamanetworkopen.2020.29676 (PMC7739119; doi:10.1001/jamanetworkopen.2020.29676)
Supplement: Supplement. — eAppendix. Defining inpatient treatment and propensity score matching approach eFigure. Comparing standardized mean difference before and after matching eTable 1. Summary of baseline and alternative propensity score matching strategies eTable 2. Cohort characteristics prior to propensity score matching eTable 3. Characteristics of unmatched individuals eTable 4. Expanded Cox proportional hazards regression model output eTable 5. Cox proportional hazards model with disaggregated medication categories eTable 6. Detailed results of 1-year outcome incidence rates eReferences [file jamanetwopen-e2029676-s001.pdf]

Supplementary Online Content

Morgan JR, Barocas JA, Murphy SM, et al. Comparison of rates of overdose and hospitalization after initiation of medication for opioid use disorder in the inpatient vs outpatient setting. *JAMA Netw Open*. 2020;3(12):e2029676. doi:10.1001/jamanetworkopen.2020.29676

eAppendix. Defining inpatient treatment and propensity score matching approach

eFigure. Comparing standardized mean difference before and after matching

eTable 1. Summary of baseline and alternative propensity score matching strategies

eTable 2. Cohort characteristics prior to propensity score matching

eTable 3. Characteristics of unmatched individuals

eTable 4. Expanded Cox proportional hazards regression model output

eTable 5. Cox proportional hazards model with disaggregated medication categories

eTable 6. Detailed results of 1-year outcome incidence rates

eReferences

This supplementary material has been provided by the authors to give readers additional information about their work.

© 2020 Morgan et al. *JAMA Network Open*.

eAppendix. Defining inpatient treatment and propensity score matching approach

Defining inpatient treatment

Inpatient treatment was categorized as either short-term inpatient or long-term residential. We defined these with revenue and HCPCS codes, and evaluated the length of stay to confirm short- and long-term categories had good internal agreement: short-term admissions had a median length of stay of 5 days and long-term admissions had a median length of stay of 14 days. The codes below are mutually exclusive and numbers are for comparing prevalence among procedures and are prior to follow up restrictions and matching that yielded the final inpatient count in the manuscript. The earliest inpatient date was taken as the initial visit. Very rarely (<1% of claims) admission to more than one facility on the same day was recorded; these admissions were dropped.

**Revenue codes**	**Code description**	n	Length of stay	
Short-term			mean	median
0116	Private medical or general-detoxification	1,695	6.3	5.0
0126	Semi-private 2 bed (med-genrl)-detoxification	58,009	6.7	5.0
0136	Semi-private 3 and 4 beds-detoxification	1,493	8.8	5.0
0146	Private (deluxe)-detoxification	7	12.7	9.0
0156	Room-Board ward (med-genrl)-detoxification	261	6.2	4.0
Long-term				
1000	Behavioral Health Room and Board (BH R & B)	120	7.2	6.0
1002	BH R & B Residential - Chem Dep	7,180	15.5	14.0
1003	BH R & B Supervised Living	7	21.7	14.0
1004	BH R & B Halfway House	7	28.7	18.0
1005	BH R & B Group Home	1	3.0	3.0

**HCPCS Procedure codes**	**Code description**	n	Length of stay	
Detoxification			mean	median
H0008	Alcohol and/or drug services; sub-acute detoxification (hospital inpatient)	50	8.9	5.5
H0009	Alcohol and/or drug services; acute detoxification (hospital inpatient)	618	7.6	5.0
H0010	Alcohol and/or drug services; sub-acute detoxification (residential addiction program inpatient)	1,113	8.8	6.0
H0011	Alcohol and/or drug services; acute detoxification (residential addiction program inpatient)	357	10.1	6.0
H0012	Alcohol and/or drug services; sub-acute detoxification (residential addiction program outpatient)	73	7.3	5.0
H0013	Alcohol and/or drug services; acute detoxification (residential addiction program outpatient)	57	6.5	4.0
H0014	Alcohol and/or drug services; ambulatory detoxification	23	6.2	4.0
Long-term				
H0017	Behavioral health; residential (hospital residential treatment program), without room and board, per diem	117	14.3	10.0
H0018	Behavioral health; short-term residential (non-hospital residential treatment program), w/o room/board, per diem	520	14.8	14.0

© 2020 Morgan et al. *JAMA Network Open*.

H0019	Behavioral health; long-term residential (non-medical, non-acute care in a residential treatment program where stay is typically longer than 30 days), w/o room and board, per diem	20	20.3	14.0

Propensity score matching approach

Propensity score matching involves several steps. The **propensity score**[1] is simply the estimated probability that an individual is assigned to a given treatment arm. In a randomized controlled trial this should be 50% (or whatever ratio of treatment to control the protocol defines), and should not vary across patients. In this study we defined "treated" cases as those initiating inpatient residential and "controls" as outpatient initiations. In observational studies like this one where treatment may not be randomly assigned, the propensity score can vary among the covariates one chooses to include in the algorithm (see the manuscript discussion for how we chose the variables). **Propensity score matching**, then, entails matching treatment to control using the propensity score to form a balanced sample. There are several decisions that go into matching. First, we must choose the matching algorithm, or how the program chooses treatment and control matches. We uses a **greedy nearest neighbor strategy**, which means that the algorithm selects a treatment observation and finds the control observation with the closest propensity score to match with. The control can only be used for a match one (this is known **as matching without replacement**). The greedy algorithm also requires the user to specify the **order** the algorithm looks for matches, descending (starting with highest treatment propensity score matching to highest control, moving down), ascending, or random. Matches from this algorithm can be poor if the propensity score of the nearest neighbor is far from that of the treatment. For this reason, we specify a **caliper** which is the maximum absolute difference in propensity scores we allow (the algorithm will match treatment to control as closely as possible). Early work suggested a caliper of 0.25[1], while more recent works suggest 0.20[2] may yield better matches without a tradeoff in sample size. Researchers employing matching should evaluate different caliper sizes. We assess the quality of the match relative to other matches by examining the **standardized mean difference** across variables. The standardized mean difference is calculated for a specific variable by subtracting the mean of the control group from the mean of the treatment group, and dividing the result by the standard deviation. Comparing the standardized mean difference before and after matching indicates how successful matching was in reducing the difference. We evaluated several different matching strategies by altering algorithm order, caliper size, and the number of controls matched to treated cases. Supplemental Table 1 below summarizes the results across these approaches. The baseline combination (caliper=0.20, random order for nearest neighbor matching, and matching outpatient controls to combined patient treatment had the best balance between low total absolute difference and high sample size, although none of the alternative matching strategies yielded statistically significantly differed treatment hazard ratios.

© 2020 Morgan et al. *JAMA Network Open*.

eFigure. Comparing standardized mean difference before and after matching

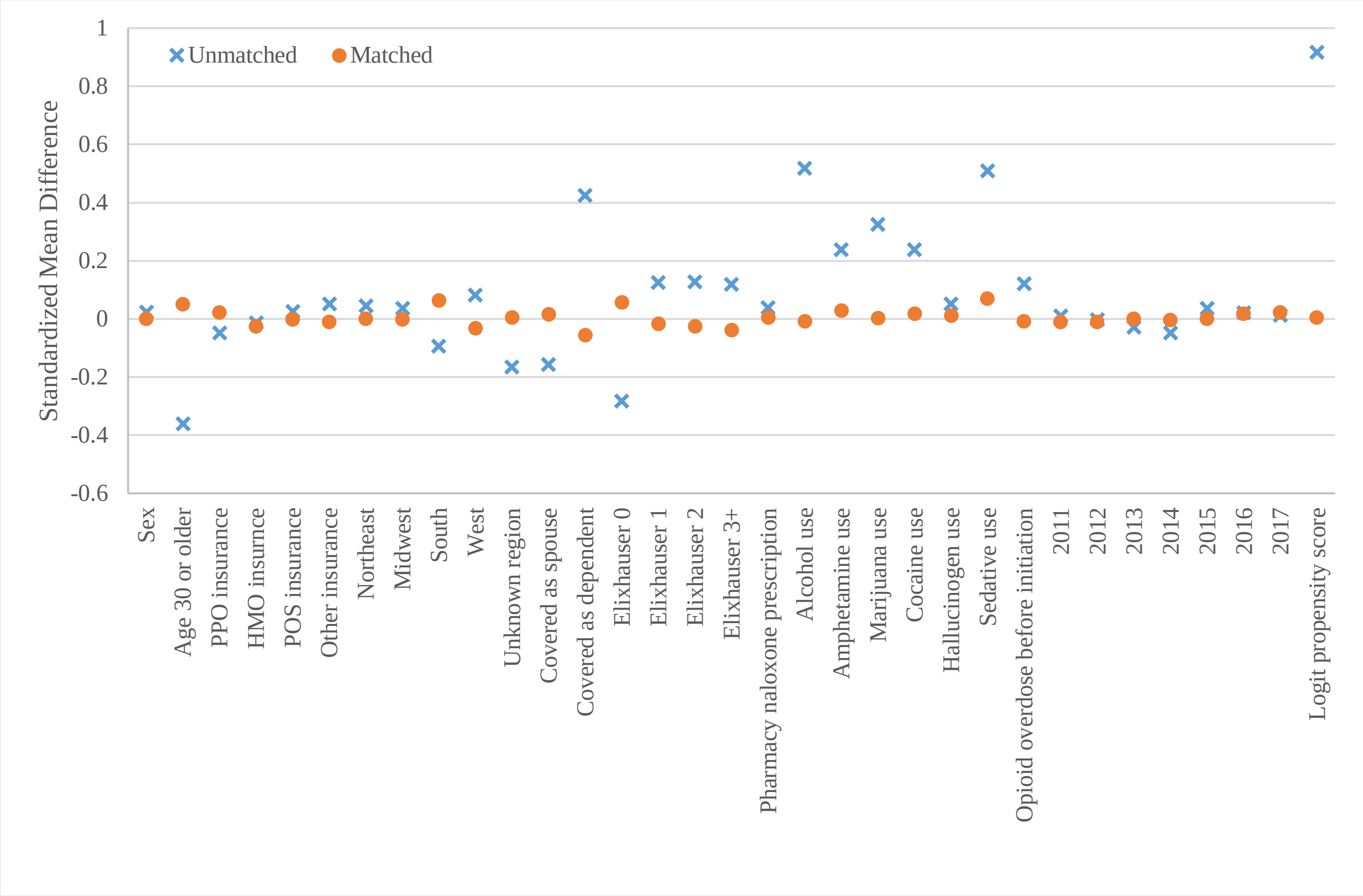

© 2020 Morgan et al. *JAMA Network Open*.

eTable 1. Summary of baseline and alternative propensity score matching strategies

Approach				Results		
Matching cohorts	Strategy	Caliper	Order	Sample size	Total absolute difference	Treatment HR point estimate outside of baseline 95% CI?
**1:1 inpatient:MOUD**	**Greedy**	**0.20**	**Random**	**37,090**	**109**	**-**
1:1 inpatient:MOUD	Greedy	0.20	Descending	38,780	1,397	No
1:1 inpatient: MOUD	Greedy	0.20	Ascending	36,600	772	No
1:1 inpatient: MOUD	Greedy	0.25	Random	37,290	152	No
1:1 inpatient: MOUD	Greedy	0.15	Random	36,924	77	No
2-level 1:1 appended<br>short-term inpatient: MOUD<br>long-term inpatient: MOUD	Greedy	0.20	Random	36,978	By level:<br>93<br>40	No
2-level 1:1 appended<br>short-term without MOUD: MOUD<br>short-term with MOUD: MOUD<br>long-term without MOUD: MOUD<br>long-term with MOUD: MOUD	Greedy	0.20	Random	61,736	By level:<br>85<br>30<br>39<br>14	No
Unmatched	-	-	-	-	-	Yes

There sample size was not large enough for robust 2:1 MOUD:inpatient matching, and attempting this yielded a total absolute difference larger than any other strategies detailed in Supplemental Table 1. The clinical expertise of our team guided our decision to match medication treatment to inpatient treatment broadly, as we believed the effect of treatment selection would be largest for the decision to initiate in inpatient vs. medication care rather than the decision to initiate short term vs. long term inpatient care. Our pre-match descriptive table below (Supplemental Table 2) also supports this with difference grouping on the inpatient vs. medication axis and less so on the individual inpatient measures. However we evaluated this by matching medication to short term and then long term inpatient treatment, and again matching separately across all four inpatient categories. To do this we randomly sorted our medication cohort, and subdivided it based on the proportions in the inpatient group: split 88%:12% for short-term (88%) vs. long-term (12%) and for the category analysis split it into sections that were 68% of total (for short-term without medication), 20% (short-term with medication, 10% long-term, 2% long-term with medication. We then conducted separate matches for each inpatient and medication subset, and appended these matched sets together for a final cohort. The results of these matching strategies are detailed in Supplemental Table 1.

Supplemental Figure 1 displays the changes in standardized mean differences. Matching substantially reduced the standardized mean difference for every variable examined. Closer to zero indicates less difference and more comparable samples – the goal of the propensity score matching procedure.

© 2020 Morgan et al. *JAMA Network Open*.

eTable 2. Cohort characteristics prior to propensity score matching

	Medication only		Inpatient		Short-term only		Short-term, medication linkage		Long-term only		Long-term, medication linkage	
	n=	*46,524*	*n=*	*24,212*	*n=*	*16,453*	*n=*	*4,619*	*n=*	*2,705*	*n=*	*435*
Sex												
Male	28,400	61.0%	15,322	63.3%	10,516	63.9%	2,788	60.4%	1,761	65.1%	257	59.1%
Female	18,124	39.0%	8,890	36.7%	5,937	36.1%	1,831	39.6%	944	34.9%	178	40.9%
Age												
<30	18,039	38.8%	13,687	56.5%	9,099	55.3%	2,429	52.6%	1,863	68.9%	296	68.0%
30 or older	28,485	61.2%	10,525	43.5%	7,354	44.7%	2,190	47.4%	842	31.1%	139	32.0%
Insurance												
PPO	29,254	62.9%	14,653	60.5%	9,928	60.3%	2,752	59.6%	1,708	63.1%	265	60.9%
HMO	5,086	10.9%	2,551	10.5%	1,812	11.0%	546	11.8%	165	6.1%	28	6.4%
POS	3,415	7.3%	1,937	8.0%	1,278	7.8%	332	7.2%	279	10.3%	48	11.0%
Other insurance	8,769	18.8%	5,071	20.9%	3,435	20.9%	989	21.4%	553	20.4%	94	21.6%
Region												
Northeast	9,700	20.8%	5,508	22.7%	4,000	24.3%	842	18.2%	580	21.4%	86	19.8%
Midwest	8,924	19.2%	5,004	20.7%	3,073	18.7%	1,389	30.1%	433	16.0%	109	25.1%
South	19,112	41.1%	8,841	36.5%	6,147	37.4%	1,533	33.2%	1,024	37.9%	137	31.5%
West	7,640	16.4%	4,741	19.6%	3,149	19.1%	823	17.8%	666	24.6%	103	23.7%
Unknown	1,148	2.5%	118	0.5%	84	0.5%	32	0.7%	2	0.1%	0	0.0%
Coverage												
Self	20,795	44.7%	7,511	31.0%	5,275	32.1%	1,509	32.7%	616	22.8%	111	25.5%
Spouse	12,252	26.3%	4,783	19.8%	3,314	20.1%	1,041	22.5%	362	13.4%	66	15.2%
Dependent	13,477	29.0%	11,918	49.2%	7,864	47.8%	2,069	44.8%	1,727	63.8%	258	59.3%
Modified Elixhauser score												
0	24,063	51.7%	9,170	37.9%	6,344	38.6%	1,630	35.3%	1,057	39.1%	139	32.0%
1	12,737	27.4%	8,013	33.1%	5,411	32.9%	1,572	34.0%	894	33.0%	136	31.3%
2	5,430	11.7%	3,887	16.1%	2,615	15.9%	769	16.6%	418	15.5%	85	19.5%
3+	4,294	9.2%	3,142	13.0%	2,083	12.7%	648	14.0%	336	12.4%	75	17.2%
Retail pharmacy naloxone prescription*												
Yes	680	1.5%	478	2.0%	265	1.6%	110	2.4%	72	2.7%	31	7.1%
No	45,844	98.5%	23,734	98.0%	16,188	98.4%	4,509	97.6%	2,633	97.3%	404	92.9%
Concurrent substance use at initiation**												
Alcohol***	3,221	6.9%	6,147	25.4%	4,185	25.4%	1,060	22.9%	761	28.1%	141	32.4%
Amphetamines	681	1.5%	1,430	5.9%	895	5.4%	174	3.8%	309	11.4%	52	12.0%
Marijuana	1,795	3.9%	3,077	12.7%	1,952	11.9%	514	11.1%	544	20.1%	67	15.4%
Cocaine	953	2.0%	1,691	7.0%	1,076	6.5%	293	6.3%	278	10.3%	44	10.1%
Hallucinogens	84	0.2%	114	0.5%	60	0.4%	13	0.3%	41	1.5%	0	0.0%
Sedatives	1,687	3.6%	4,694	19.4%	3,319	20.2%	765	16.6%	522	19.3%	88	20.2%
1+ Overdose before initiation**												
Yes	417	0.9%	592	2.4%	380	2.3%	117	2.5%	78	2.9%	17	3.9%
No	46,107	99.1%	23,620	97.6%	16,073	97.7%	4,502	97.5%	2,627	97.1%	418	96.1%

*Any time from 90 days before treatment initiation

**Diagnosis code in 90 days before first initiation

***We exclude oral naltrexone initiations where AUD is present within 90 days prior

© 2020 Morgan et al. *JAMA Network Open*.

eTable 3: Characteristics of unmatched individuals

	Unmatched who initiated with medication treatment		Unmated who initiated with inpatient care		Chi-square p-value
	n=27,979		*n=5,667*		
Sex					
Male	16,775	60.0%	3,697	65.2%	<0.01
Female	11,204	40.0%	1,970	34.8%	
Age					
<30	7,448	26.6%	3,555	62.7%	<0.01
30 or older	20,531	73.4%	2,112	37.3%	
Insurance					
PPO	18,185	65.0%	3,372	59.5%	<0.01
HMO	2,924	10.5%	527	9.3%	
POS	1,944	6.9%	469	8.3%	
Other insurance	4,926	17.6%	1,299	22.9%	
Region					
Northeast	5,128	18.3%	1,274	22.5%	<0.01
Midwest	5,101	18.2%	1,194	21.1%	
South	12,805	45.8%	1,967	34.7%	
West	3,901	13.9%	1,230	21.7%	
Unknown	1,044	3.7%	2	0.0%	
Insurance Coverage					
Primary holder	15,090	53.9%	1,442	25.4%	<0.01
Spouse	8,593	30.7%	991	17.5%	
Dependent	4,296	15.4%	3,234	57.1%	
Modified Elixhauser score*					
0	16,556	59.2%	1,146	20.2%	<0.01
1	6,881	24.6%	2,290	40.4%	
2	2,587	9.2%	1,206	21.3%	
3+	1,955	7.0%	1,025	18.1%	
Retail pharmacy naloxone prescription**					
Yes	349	1.2%	134	2.4%	<0.01
No	27,630	98.8%	5,533	97.6%	
Concurrent substance use at initiation***					
Alcohol****	31	0.1%	3,004	53.0%	<0.01
Amphetamines	43	0.2%	693	12.2%	<0.01
Marijuana	129	0.5%	1,398	24.7%	<0.01
Cocaine	82	0.3%	746	13.2%	<0.01
Hallucinogens	25	0.1%	42	0.7%	<0.01
Sedatives	18	0.1%	2,625	46.3%	<0.01
1+ Overdose before initiation***					
Yes	62	0.2%	254	4.5%	<0.01
No	27,917	99.8%	5,413	95.5%	
*Modified to exclude alcohol and drug use<br>*Any time from 90 days before treatment initiation					
**Diagnosis code in 90 days before first initiation					

© 2020 Morgan et al. *JAMA Network Open*.

***We exclude oral naltrexone initiations where AUD is present within 90 days prior	

eTable 4. Expanded Cox proportional hazards regression model output

	**Opioid-related overdose**	**All-cause hospitalization**
	Hazard ratio (95% Confidence interval)	Hazard ratio (95% Confidence interval)
Treatment initiation		
Medication	Reference	Reference
Short-term inpatient	2.23 (1.97-2.52)	1.90 (1.83-1.97)
Short-term inpatient followed by medication	2.08 (1.75-2.47)	1.74 (1.64-1.84)
Long-term inpatient	1.71 (1.35-2.17)	1.33 (1.23-1.44)
Long-term inpatient followed by medication	2.67 (1.68-4.23)	1.16 (0.96-1.42)
Sex		
Male	Reference	Reference
Female	0.95 (0.85-1.07)	1.18 (1.14-1.22)
Age		
<30	Reference	Reference
30 or older	0.49 (0.38-0.63)	0.87 (0.80-0.93)
Insurance		
PPO	Reference	Reference
HMO	1.25 (1.07-1.47)	0.95 (0.90-1.00)
POS	0.99 (0.81-1.21)	1.01 (0.95-1.08)
Other insurance	1.26 (1.10-1.44)	1.06 (1.01-1.10)
Region		
Northeast	Reference	Reference
Midwest	1.15 (1.00-1.33)	0.87 (0.83-0.91)
South	0.65 (0.56-0.75)	0.83 (0.79-0.87)
West	0.61 (0.51-0.72)	0.83 (0.79-0.87)
Unknown	0.72 (0.34-1.51)	0.70 (0.55-0.91)
Insurance Coverage		
Primary holder	Reference	Reference
Spouse	0.84 (0.67-1.05)	1.12 (1.06-1.18)
Dependent	1.65 (1.30-2.08)	1.51 (1.41-1.63)
Modified Elixhauser score*		
0	Reference	Reference
1	1.06 (0.93-1.20)	1.13 (1.09-1.18)
2	1.32 (1.11-1.57)	1.36 (1.29-1.44)
3+	1.45 (1.18-1.79)	1.94 (1.83-2.06)
Retail pharmacy naloxone prescription		
Yes	1.95 (1.51-2.52)	1.74 (1.57-1.92)
No	Reference	Reference
Concurrent substance use at initiation**		
Alcohol***	0.91 (0.77-1.08)	1.11 (1.06-1.17)
Amphetamines	0.99 (0.73-1.35)	1.14 (1.04-1.25)

© 2020 Morgan et al. *JAMA Network Open*.

Marijuana	0.93 (0.79-1.10)	1.01 (0.95-1.07)
Cocaine	1.28 (1.02-1.61)	1.19 (1.11-1.29)
Hallucinogens	0.90 (0.40-2.01)	1.12 (0.87-1.44)
Sedatives	1.30 (1.09-1.54)	1.11 (1.05-1.17)
1+ Overdose before initiation		
Yes	3.29 (2.64-4.09)	1.21 (1.08-1.35)
No	Reference	Reference
Year of initiation		
2011	Reference	Reference
2012	0.82 (0.69-0.98)	0.92 (0.87-0.97)
2013	1.07 (0.90-1.27)	0.92 (0.87-0.98)
2014	1.04 (0.87-1.24)	0.95 (0.90-1.01)
2015	0.87 (0.72-1.05)	0.97 (0.91-1.03)
2016	0.78 (0.62-0.98)	0.91 (0.85-0.97)
2017	0.66 (0.48-0.92)	1.02 (0.94-1.11)

Each model simultaneously adjusts for all included variables. We also controlled for region of residence, type of commercial insurance, and year of initiation. Full results available in the supplemental appendix. Sex, age, and insurance coverage are measured at time of initial treatment. Elixhauser, pharmacy naloxone, concurrent substance use, and overdose prior to initiation are measured using data 90 days prior to initiation.
*Elixhauser score is modified to exclude drug and alcohol use as those are included separately
**Substance use variables are dichotomous (reference is no evidence of use) and overlapping
***Those with evidence of alcohol use disorder were not eligible for inclusion in the oral naltrexone cohort

© 2020 Morgan et al. *JAMA Network Open*.

Results of E-Value analysis

E-values were large compared to the included model confounders for both outcomes. For overdose, the E-values (confidence limit) for inpatient treatment were 3.60 (2.95), 3.56 (3.08), 4.80 (3.10), and 3.00 (2.24) for short-term inpatient with and without MOUD to follow and long-term inpatient with and without MOUD to follow, respectively. For the same exposure groups in the all-cause hospitalization outcome, the corresponding values were 2.23 (2.11), 2.34 (2.27), 1.25 (1.00), and 1.40 (1.20). This suggests that residual confounding could explain the observed association if there exists an unmeasured covariate having a relative risk association at least as large as 4.80 for the overdose model and 2.34 for the all-cause hospitalization model.

© 2020 Morgan et al. *JAMA Network Open*.

eTable 5. Cox proportional hazards model with disaggregated medication categories

	**Opioid-related overdose**	**All-cause hospitalization**
	Hazard ratio (95% Confidence interval)	Hazard ratio (95% Confidence interval)
Treatment initiation		
Buprenorphine	Reference	Reference
Oral naltrexone	1.33(0.99-1.78)	1.10 (1.00-1.22)
Injectable naltrexone	0.81(0.52-1.26)	1.04 (0.94-1.16)
Short-term inpatient	2.27 (2.00-2.58)	1.92 (1.85-2.00)
Short-term inpatient followed by MOUD	2.12 (1.77-2.53)	1.76 (1.66-1.86)
Long-term inpatient	1.74 (1.37-2.22)	1.34 (1.24-1.46)
Long-term inpatient followed by MOUD	2.72 (1.71-4.33)	1.18 (0.97-1.43)
Sex		
Male	Reference	Reference
Female	0.95 (0.85-1.07)	1.18 (1.14-1.22)
Age		
<30	Reference	Reference
30 or older	0.49 (0.38-0.63)	0.87 (0.80-0.93)
Insurance		
PPO	Reference	Reference
HMO	1.25 (1.07-1.47)	0.95 (0.90-1.00)
POS	0.99 (0.81-1.21)	1.01 (0.95-1.08)
Other insurance	1.25 (1.10-1.43)	1.06 (1.01-1.10)
Region		
Northeast	Reference	Reference
Midwest	1.16 (1.00-1.33)	0.87 (0.83-0.91)
South	0.65 (0.56-0.75)	0.83 (0.79-0.87)
West	0.61 (0.51-0.72)	0.83 (0.79-0.88)
Unknown	0.72 (0.34-1.52)	0.71 (0.55-0.91)
Insurance Coverage		
Primary holder	Reference	Reference
Spouse	0.84 (0.67-1.05)	1.12 (1.06-1.18)
Dependent	1.64 (1.30-2.07)	1.51 (1.40-1.62)
Modified Elixhauser score*		
0	Reference	Reference
1	1.05 (0.92-1.19)	1.13 (1.08-1.18)
2	1.31 (1.10-1.56)	1.36 (1.29-1.44)
3+	1.44 (1.17-1.77)	1.93 (1.82-2.05)
Retail pharmacy naloxone prescription		
Yes	1.94 (1.50-2.51)	1.74 (1.57-1.92)
No	Reference	Reference
Concurrent substance use at initiation**		

© 2020 Morgan et al. *JAMA Network Open*.

Alcohol***	0.94 (0.79-1.11)	1.11 (1.06-1.17)
Amphetamines	0.98 (0.72-1.34)	1.14 (1.04-1.24)
Marijuana	0.92 (0.78-1.09)	1.00 (0.95-1.06)
Cocaine	1.28 (1.02-1.60)	1.19 (1.10-1.28)
Hallucinogens	0.89 (0.40-2.00)	1.12 (0.87-1.43)
Sedatives	1.29 (1.09-1.53)	1.10 (1.04-1.17)
1+ Overdose before initiation		
Yes	3.27 (2.63-4.07)	1.20 (1.08-1.35)
No	Reference	Reference
Year of initiation		
2011	Reference	Reference
2012	0.82 (0.69-0.98)	0.92 (0.87-0.97)
2013	1.07 (0.90-1.27)	0.92 (0.87-0.97)
2014	1.04 (0.87-1.24)	0.95 (0.90-1.01)
2015	0.87 (0.72-1.05)	0.97 (0.91-1.03)
2016	0.78 (0.62-0.97)	0.91 (0.85-0.97)
2017	0.66 (0.48-0.92)	1.02 (0.94-1.11)

Each model simultaneously adjusts for all included variables. Sex, age, and insurance coverage are measured at time of initial treatment. Elixhauser, pharmacy naloxone, concurrent substance use, and overdose prior to initiation are measured using data 90 days prior to initiation.
* Modified to exclude drug and alcohol use
**Substance use variables are dichotomous (reference is no evidence of use) and overlapping
***Those with evidence of alcohol use disorder were not eligible for inclusion in the oral naltrexone cohort

© 2020 Morgan et al. *JAMA Network Open*.

eTable 6. Detailed results of 1-year outcome incidence rates

**Cohort**	**Outcome**	**1-year count**	**1-year person-years**	**Rate/100PY**	**95% CI**
MOUD only *n=18,545*	Opioid-related overdoses	415	18,545	2.24	(2.03-2.46)
	All cause hospitalizations	7,258	18,545	39.14	(38.25-40.05)
Residential, MOUD linkage *n=313*	Opioid-related overdoses	22	313	7.03	(4.62-10.67)
	All cause hospitalizations	196	313	62.62	(54.44-72.03)
Detox, MOUD linkage *n=3,683*	Opioid-related overdoses	135	3,683	3.67	(3.10-4.34)
	All cause hospitalizations	2,342	3,683	63.59	(61.59-66.22)
Residential only *n=1,921*	Opioid-related overdoses	67	1,921	3.49	(2.74, 4.43)
	All cause hospitalizations	1,102	1,921	57.37	(54.08-60.85)
Detox only *n=12,628*	Opioid-related overdoses	544	12,628	4.31	(3.96-4.69)
	All cause hospitalizations	9,354	12,628	74.07	(72.59-75.59)

© 2020 Morgan et al. *JAMA Network Open*.

eReferences

1. Rosenbaum PR, Rubin DB. Constructing a Control Group Using Multivariate Matched Sampling Methods That Incorporate the Propensity Score. The American Statistician 1985;39:33.

2. Austin PC. Optimal caliper widths for propensity-score matching when estimating differences in means and differences in proportions in observational studies. Pharmaceutical Statistics 2011;10:150-61.

© 2020 Morgan et al. *JAMA Network Open*.
